# Development of a recombinant membrane protein ELISA for analyzing antibody responses against SARS-CoV-2 envelope proteins

**DOI:** 10.1016/j.jbc.2025.110974

**Published:** 2025-11-25

**Authors:** Sylvie Chabot, Tahir Malik, Lila Saye, Martina Kosikova, Hyeog Kang, Zhiping Ye, Matthew Memoli, Marian Major, Jan-Willem de Gier, Robert Daniels

**Affiliations:** 1 Division of Viral Products, Center for Biologics Evaluation and Research, Food and Drug Administration, Silver Spring, Maryland, USA; 2 Laboratory of Infectious Diseases Clinical Studies Unit, Laboratory of Infectious Diseases, Division of Intramural Research, National Institute of Allergy and Infectious Diseases, National Institutes of Health, Bethesda, Maryland, USA; 3 Department of Biochemistry and Biophysics, Stockholm University, Stockholm, Sweden

**Keywords:** enveloped virus surface antigens, COVID-19, spike (S) antigen, membrane (M) protein, envelope (E) protein, human antibody responses, immunoglobulin (IgG) measurements, membrane protein purification, coronavirus

## Abstract

Recombinant surface antigens from enveloped viruses are commonly produced without the transmembrane domains to avoid difficulties associated with membrane protein expression and purification. Here, we overcame these challenges by fusing several SARS-CoV-2 envelope proteins to green fluorescent protein and a Strep-tag to facilitate protein detection and purification. This approach simplified the insect cell expression optimization and purification of recombinant prefusion stabilized full-length spike (rS-GFP), membrane (rM-GFP) and envelope (rE-GFP) proteins from the ancestral SARS-CoV-2 strain. We then used the isolated membrane proteins to develop an Enzyme-linked immunosorbent assay that leverages the Strep-tag for proper antigen display and GFP fluorescence for coating consistency. Initial tests with SARS-CoV-2 patient samples from 2020 (n = 68) and healthy adult volunteer samples from 2022-24 (n = 19) revealed a range of rS-GFP reactivity but minimal rM-GFP and rE-GFP reactivity. Approximately 55% of the 2020 patient samples were S-positive compared to ∼85% of the more recent healthy volunteer samples, suggesting the more recent healthy individuals had increased exposure to SARS-CoV-2 antigens by infection or vaccination. Recombinant S (rS) without the green fluorescent protein yielded similar results and detected more S-positive samples than the recombinant receptor binding domain alone. Finally, we confirmed all the S-positive samples neutralized S-pseudotyped particles and found that the enzyme-linked immunosorbent assay data from rS and recombinant receptor binding domain can be combined to identify high and low neutralization titer samples. The results from this proof of concept study suggest a similar strategy could be applied to rapidly develop assays for other enveloped virus surface antigens.

Enzyme-linked immunosorbent assays (ELISAs) are widely used for measuring antibodies against viral antigens because of the simplicity, sensitivity, and cost-effectiveness (1). For diagnostic ELISAs one of the most critical parameters is the specificity of the antigen, whereas ELISAs designed for assessing the presence of functional antibodies are highly dependent on the antigen composition, conformation, and presentation. Commonly, ELISA antigens are either captured and displayed by antibodies adsorbed to the plate (*i.e.,* sandwich ELISA) or directly adsorbed to the plate (2). While direct adsorption involves fewer steps, sandwich ELISAs minimize antigen interactions with the plate surface which can potentially mask epitopes or alter antigen conformations (3, 4, 5). The use of purified viruses for some ELISAs can alleviate concerns about the antigen conformation (6, 7, 8), but not all viruses are easily propagated (9, 10, 11), some have strict biocontainment requirements (12) and this approach limits the ability to assign antibody responses to a specific antigen. To overcome these issues, ELISAs are typically performed with purified recombinant viral proteins.

Enveloped viruses are surrounded by a membrane that is decorated with viral envelope proteins essential for infection (13). Many of these envelope proteins serve as targets for neutralizing antibodies and all of them are embedded in the viral membrane by one or more transmembrane (TM) domain. However, antibody responses against viral envelope proteins are commonly measured with recombinant versions lacking the TM domains to avoid the difficulties associated with expressing and isolating membrane proteins. The designs of these recombinant viral envelope proteins significantly vary, ranging from the entire ectodomain (5, 14) to smaller individual domains (15) and peptides (16, 17, 18). While these recombinant approaches have been sufficient for many well-characterized viral surface antigens, most have relied on structural and empirical data to guide the design.

The main envelope proteins on the surface of the coronavirus SARS-CoV-2 are the spike (S), membrane (M) and envelope (E) proteins (19, 20). During the initial phase of the SARS-CoV-2 pandemic, an ELISA for measuring antibodies against the trimeric S protein fusogen was quickly established (21). This assay used a secreted prefusion stabilized version of the S ectodomain that lacked the TM domain and included two stabilizing modifications that were previously developed using an S protein from a related coronavirus (22). Since then, an ELISA using recombinant full-length S has not been established and linear peptides are still used for monitoring antibody responses to M and E (16, 17, 18, 23). This highlights the need for standard approaches to produce recombinant full-length envelope proteins as TM domains can influence their conformation (24, 25, 26, 27, 28) and full-length antigens are likely to provide a more complete characterization of antibody responses in sera.

Many advances have been made for optimizing membrane protein expression, purification and characterization. These range from using the green fluorescent protein (GFP) for detection to the development of new affinity tags for purification (29, 30). Here, we applied some of these strategies to optimize the insect cell expression and isolation of the full-length envelope proteins S, M and E from the ancestral SARS-CoV-2 strain (Wuhan/Hu-1/2020). We then established an ELISA that utilized the Strep-tag to properly display the viral envelope proteins and the GFP fluorescence to monitor plate binding consistency. Our results from two human sample panels showed a range of S reactivity, but only marginal M and E reactivity. The ELISA with full-length prefusion stabilized S identified more S-positive samples than with the recombinant receptor binding domain (rRBD) alone, and all of them neutralized S-pseudotyped particles, indicating it generated fewer false negatives. Interestingly, we found that the ELISA data with rS and RBD can be combined to distinguish between samples with high and low neutralization titers. These results suggest a similar strategy could be applied to rapidly develop assays for surface antigens from other enveloped viruses.

## Results

### Optimization of recombinant full-length S, M and E expression in Sf9 cells

**Figure 1:**
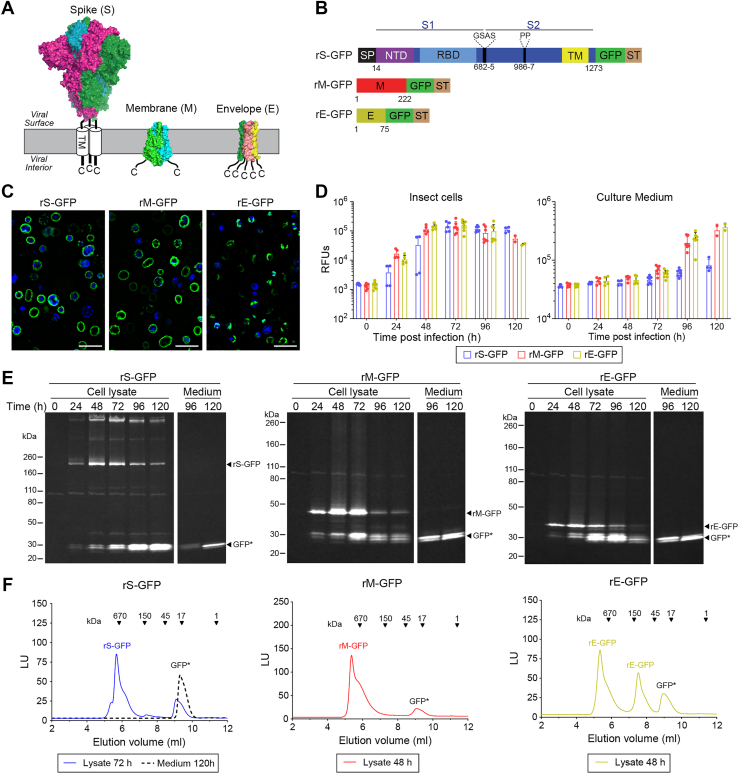
**Expression optimization of recombinant GFP-tagged SARS-CoV-2 envelope proteins.***A,* schematic of the SARS-CoV-2 Spike [S; PDB:7DDD (55)], Membrane [M; PDB 7VGR (38)] and Envelope [E; PDB: 7K3G (37)] protein topologies in the viral envelope. All structures were generated with PyMOL. *B,* diagrams of the rS-GFP, rM-GFP, and rE-GFP constructs from the Wuhan/Hu-1/2020 strain that were inserted into BV for insect cell expression. Positions of the signal peptide (SP), GFP, Strep-tag (ST), transmembrane (TM) domain and prefusion stabilizing S mutations are indicated. *C,* fluorescence microscopy images of BV-infected insect cells expressing rS-GFP (72 h post infection), rM-GFP (48 h post-infection) and rE-GFP (48 h post-infection). Bar = 50 μm. *D,* Relative fluorescence units (RFUs) from Sf9 cells and culture medium at the indicated times post-infection with BVs expressing rS-GFP, rM-GFP and rE-GFP are displayed. Each circle represents an independent biological replicate result. *E,* Representative in-gel fluorescence images showing rS-GFP, rM-GFP and rE-GFP integrity at the indicated times post-infection. Cell lysates or clarified culture medium were resolved by SDS-PAGE gel (8–16%) prior to imaging. GFP∗ denotes cleaved GFP. *F,* Representative fluorescent size exclusion chromatography (FSEC) of rS-GFP, rM-GFP, and rE-GFP in solubilized cell lysates and medium at the indicated times post-infection are displayed. Luminescence units and molecular weight standards are included for reference. Peaks corresponding to the full-length proteins and GFP∗ are indicated. GFP, green fluorescent protein.

We initiated this study to establish a standard approach for producing recombinant viral envelope proteins with intact TM domains that can be used for measuring antibody responses in an ELISA. As a proof of concept, we chose the spike (S), membrane (M), and envelope (E) proteins from SARS-CoV-2, which are all homo-oligomers with one or more TM domains (Fig. 1*A*). To begin we generated baculoviruses (BVs) that express recombinant prefusion stabilized full-length S (rS-GFP), M (rM-GFP) and E (rE-GFP) proteins with a C-terminal monomeric GFP for detection and a Strep-tag for purification (Fig. 1*B*). Following BV infection, rS-GFP and rM-GFP fluorescence predominantly accumulated at the insect cell periphery, suggesting both proteins were properly targeted to the secretory pathway and trafficked to the plasma membrane (Fig. 1*C*). In contrast, rE-GFP fluorescence localized to discrete regions in the cell which is in line with previous reports showing E accumulates in the endoplasmic reticulum-*Golgi* network when it is expressed by itself (31, 32, 33).

Next, we utilized GFP fluorescence for optimizing expression. rM-GFP and rE-GFP expression typically peaked in cells 48 to 72 h post-infection and then decreased, whereas rS-GFP expression peaked at 72 h and remained relatively steady (Fig. 1*D*). The decrease in fluorescence from rM-GFP and rE-GFP in cells coincided with increased fluorescence in the culture medium, suggesting GFP had been cleaved (Fig. 1*D*). Therefore, we examined the protein integrity by SDS-PAGE in-gel fluorescence (29, 34) and by fluorescent size exclusion chromatography (FSEC) (35). In-gel fluorescence of cell lysates confirmed that the optimal expression time for full-length prefusion stabilized rS-GFP is ∼72 h in our system, while peak expression of the smaller rM-GFP and rE-GFP proteins is ∼48 h (Fig. 1*E*). FSEC data supported this conclusion as the high molecular weight profiles of these proteins in cell lysates showed the strongest fluorescence intensity at these times (Figs. 1*F* and S1). Both assays also indicated that the GFP on all three proteins is likely susceptible to proteolytic cleavage as a band and peak corresponding to monomeric GFP (∼25 kDa) became more pronounced with time and accumulated in the medium (Figs. 1, *E* and *F* and S1).

### Development of a purification scheme for recombinant full-length S, M and E proteins

**Figure 2:**
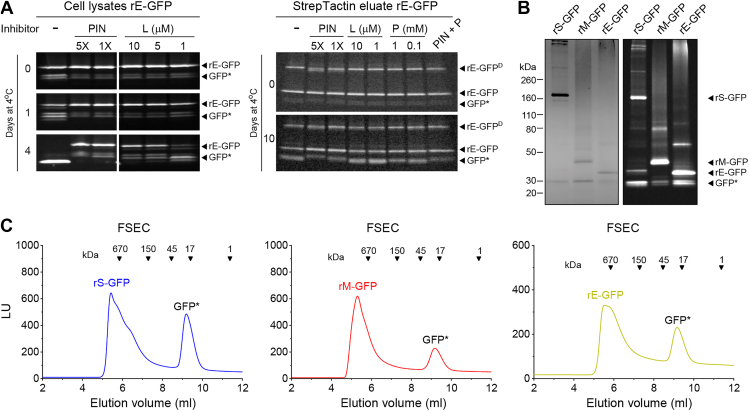
**Purification optimization of recombinant GFP-tagged S, M and E from SARS-CoV-2.***A,* representative in-gel (8–16%) fluorescence images showing rE-GFP integrity in cell lysates (*left panels*) and a crude StrepTactin eluate (*right panels*) after the indicated times at 4 °C with or without addition of a protease inhibitor (PIN) cocktail, leupeptin (L), PMSF (P) or PIN + P. rE-GFP^D^ denotes SDS resistant rE-GFP dimers. *B,* representative Coomassie (*left panel*) and in-gel fluorescence (*right panel*) images of purified rS-GFP, rM-GFP and rE-GFP (∼2.5 μg) resolved by SDS-PAGE (8–16% gel). *C,* representative FSEC chromatograms of purified rS-GFP, rM-GFP and rE-GFP proteins are displayed. Molecular weight standards are included for reference. Peaks corresponding to the full-length proteins and GFP∗ are indicated.

Since we planned to use the Strep-tag for purification and it is positioned after the GFP, we initially tested if a common Protease Inhibitor (PIN) cocktail could prevent rE-GFP cleavage in whole cell lysates by an SDS-PAGE in-gel fluorescent assay (Fig. 2*A*, left panel). In the presence of 1× and 5× PIN, full-length rE-GFP remained the dominant fluorescent band even after 4 days at 4 °C. In contrast, cleaved GFP was the only visible band in the untreated lysates after 4 days. Addition of leupeptin alone, a component of the PIN cocktail, largely retained full-length rE-GFP, indicating an insect cell cysteine or serine protease is likely responsible for the cleavage. As proteases can potentially co-purify with proteins, we also screened inhibitors in a crude rE-GFP eluate (Fig. 2*A*, right panel). After 10 days at 4 °C, more full-length rE-GFP was retained when PIN or the irreversible serine protease inhibitor phenylmethyl sulfonyl fluoride (PMSF) were added, but not leupeptin, suggesting rE-GFP may be susceptible to cleavage by more than one insect cell protease.

Following these results, we developed a single purification scheme with a cell resuspension buffer and a lysis buffer that contained PIN and PMSF (Fig. S2). Insect cells were harvested ∼48 h (rE-GFP and rM-GFP) or 72 h (rS-GFP) post-infection by sedimentation, resuspended in buffer, emulsified, and the membrane fraction was isolated by sedimentation. The sedimented membranes were solubilized using lysis buffer containing 2% (v/v) of the detergent Triton X-100 (TX-100), insoluble material was removed, and the proteins were isolated from the clarified supernatant using StrepTactin resin. The purified proteins were resolved by SDS-PAGE and visualized by in-gel fluorescence and Coomassie staining to assess purity and integrity. In both analyses we observed a dominant band, corresponding to 40 to 60% of the total protein, which migrated at the expected molecular weight of each full-length protein (Fig. 2*B*) and a lower band that corresponded to cleaved GFP (GFP∗). FSEC profiles showed that the full-length purified proteins primarily formed high molecular weight species in 0.01% TX-100, indicative of oligomer formation, whereas the cleaved GFP eluted as a monomer (Fig. 2*C*).

### Development of a fluorescence-based coating ELISA for GFP-tagged S, M and E

**Figure 3:**
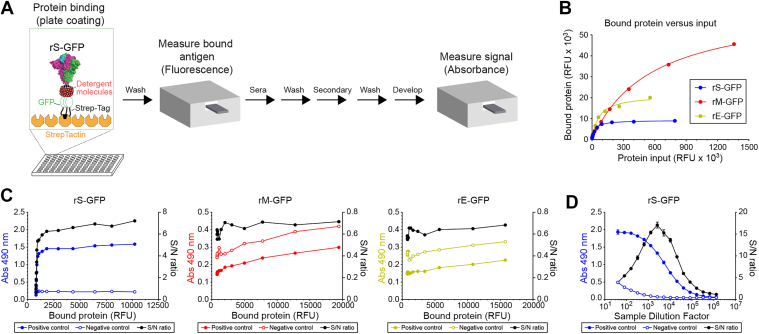
**Development of an ELISA using purified recombinant GFP-tagged S, M and E.***A,* ELISA methodology for monitoring bound rS-GFP, rM-GFP and rE-GFP amounts. *B,* representative binding curves of rS-GFP, rM-GFP and rE-GFP to StrepTactin-coated wells. Serially diluted proteins were added to the plate and relative fluorescence units (RFUs) were recorded. Plates were incubated 2 h at 37 °C, washed and the bound RFUs measured. Results are the mean from an experiment performed in duplicate. *C,* signal to noise (S/N) ratios are displayed from an ELISA performed with fixed dilutions (1:250) of a pooled human SARS-CoV-2 positive sample and a negative human pre-SARS-CoV-2 sample in wells with the indicated bound amounts of rS-GFP, rM-GFP and rE-GFP. Results are the mean from a representative experiment performed in duplicate. *D,* S/N ratios are displayed from an ELISA performed with serial dilutions of the human SARS-CoV-2 positive sample and negative control in wells with a fixed amount of bound rS-GFP (∼2000 RFUs or ∼40 ng). Results are the mean from an experiment performed in duplicate.

Experimental and predictive data indicates that the S, M and E proteins all have an intracellular *C*-terminus (36, 37, 38). Therefore, we established an ELISA where we used StrepTactin-coated plates to display each recombinant protein by the *C*-terminal Strep-tag and fluorescence to determine the bound protein amounts based on a standard curve for each recombinant protein (Figs. 3*A* and S3). Titrations with rS-GFP and rE-GFP showed that binding saturation was reached at ∼9000 (∼250 ng) and ∼18,000 (∼500 ng) bound relative fluorescence unit (RFUs), respectively (Fig. 3*B*). However, rM-GFP did not reach saturation even at undiluted input amounts, suggesting M likely forms higher order oligomers that bind to itself as previously reported (38, 39).

To determine bound protein amounts that resulted in the highest signal/noise (S/N) ratio we used fixed dilutions (1:250) of a positive human SARS-CoV-2 sample (WHO standard) and a pre-SARS-CoV-2 negative human sample (Fig. 3*C*). The S/N ratio from the positive control increased with the amount of bound rS-GFP and reached a plateaued at ∼2000 RFUs (∼40 ng) of bound rS-GFP (Fig. 3*C*, left). In contrast, no S/N increase was observed with rM-GFP and rE-GFP and the negative control was slightly higher than the positive (Fig. 3*C*, middle and right). Similar results were observed with SARS-CoV-2 positive and negative mouse sera (Fig. S4), indicating M and E are either not very immunogenic or the rM-GFP and rE-GFP are not properly folded. Based on these observations, we determined the optimal sample dilution by titrating the human positive and negative controls using a fixed amount (∼2000 RFUs or 40 ng) of bound rS-GFP (Fig. 3*D*). With this fixed amount of rS-GFP the S/N ratio showed a clear peak at a 1:2000 dilution and the positive signal was still evident at dilutions several orders of magnitude higher, indicating 1:2000 could be a suitable dilution for a larger sample panel.

### Sera from SARS-CoV-2 patients strongly react with rS-GFP but not rM-GFP or rE-GFP

**Figure 4:**
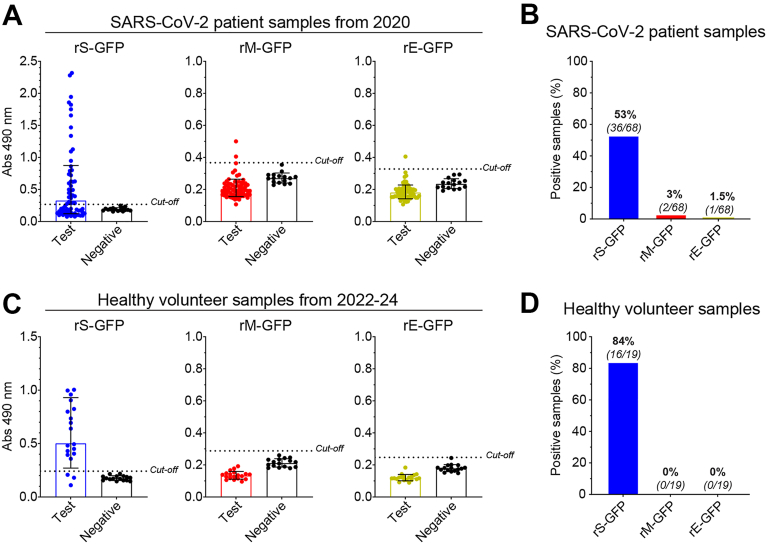
**Reactivity of SARS-CoV-2 positive and healthy volunteer sera with rS-GFP, rM-GFP and rE-GFP.***A,* ELISA results are displayed for a panel of SARS-CoV-2 patient samples from 2020 (n = 68) and a panel of human pre-SARS-CoV-2 samples (n = 16). Samples diluted 1/2000 were run in duplicate and the mean Absorbance (Abs) at 490 nm is displayed. Positive signal cut-off (*dashed line*) corresponds to the mean of the pre-SARS-CoV-2 samples plus 3 SDs. *B,* percentage of the SARS-Cov-2 patient samples from 2020 that reacted with rS-GFP, rM-GFP and rE-GFP are displayed. Positive samples possessed an Abs 490 nm greater than the positive signal cut-off of the negative control samples. *C,* ELISA results are displayed for a panel of human healthy volunteer sera (n = 19) from 2022 to 2024 and the pre-SARS-CoV-2 negative control samples (n = 16). Samples diluted 1/2000 were run in duplicate and the mean Abs at 490 nm is displayed. *Dashed lines* indicate the positive signal cut-off. *D,* percentage of the human healthy volunteer samples that reacted with rS-GFP, rM-GFP and rE-GFP are displayed. Positive samples possessed a mean Abs 490 nm greater than the positive signal cut-off of the negative control samples. ELISA, enzyme-linked immunosorbent assay.

To test the identified ELISA parameters we used a panel (n = 68) of SARS-CoV-2 patient samples from 2020 and a negative control group (n = 16) of pre-SARS-CoV-2 samples from 2013 to 2014. The patient samples displayed a wide range of rS-GFP reactivity (Fig. 4*A*, left) and the absorbance values correlated with those from an independent lab that used lower sample dilutions and plates coated with a secreted S protein produced in Expi293F cells (Fig. S5). In contrast, rM-GFP and rE-GFP responses were largely similar to the controls (Fig. 4*A*, middle and right), in line with early reports that M and E are less immunogenic than S (18, 40). However, it is also possible that the rM-GFP and rE-GFP used in our assay are not presented in their native conformation.

To identify ELISA positive samples, we used the mean of the negative control samples plus three SDs. With this approach, 36 test samples (53%) were found to be reactive against rS-GFP, 2 (3%) against rM-GFP and 1 (1.5%) against rE-GFP (Fig. 4*B*). This suggests that the SARS-CoV-2 patients from 2020 predominantly elicited anti-S antibodies and that some individuals did not generate measurable antibody responses, in line with previous analyses of SARS-CoV-2 patient sera from 2020 to 2021 (41, 42). To examine if antibody response frequencies against S, M and E changed over time, we tested a panel (n = 19) of healthy volunteer sera collected between 2022 and 2024 (Fig. 4*C*). These patients were not pre-screened for SARS-CoV-2 and 16 out of 19 samples (84%) reacted with rS-GFP from the ancestral Wuhan strain, but none reacted with rM-GFP or rE-GFP (Fig. 4*D*). These results suggest that a high percentage of the healthy adults possess S-reactive antibodies likely due to increased exposure to SARS-CoV-2 antigens by infection or vaccination.

### Comparison of human sera reactivity with full-length S, secreted S and RBD

**Figure 5:**
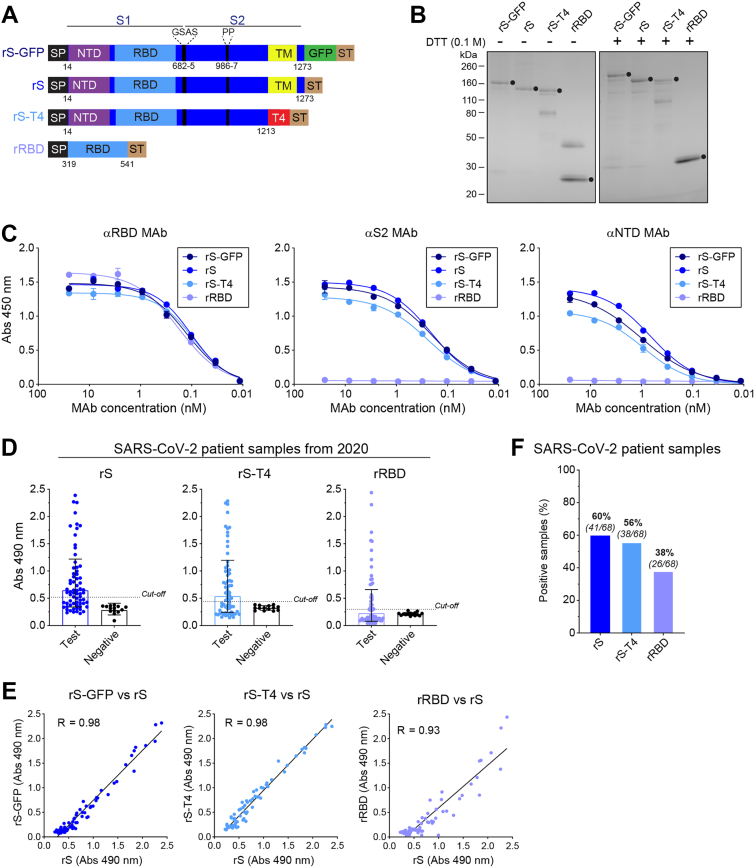
**Comparison of ELISA results with recombinant full-length and soluble S proteins.***A,* schematics of the BV expression constructs encoding full-length S from the Wuhan/Hu-1/2020 strain without GFP (rS), a secreted S with a T4 trimerization domain in place of the TM domain (rS-T4), and the RBD. rS-GFP is shown for comparison. Positions of the SP, GFP, ST, T4 and the prefusion stabilizing mutations are indicated. *B,* representative Coomassie stained SDS-PAGE gel (8–16%) showing the purified rS-GFP, rS, rS-T4 and RBD (*circles*). Samples were mixed with sample buffer and loaded directly or with sample buffer containing 0.1 M dithiothreitol and heated at 95 °C for 5 min prior to loading. *C,* ELISA titration curves showing the binding of MAbs against the RBD (*left*), S2 (*cente*r) and NTD (*right*) to rS-GFP, rS, rS-T4 and rRBD. MAbs (25 nM) were serially diluted three-fold and added to wells coated with a fixed amount (∼2000 RFU) of the indicated recombinant protein. Samples were run in duplicate, and the mean Abs 450 nm is shown. *D,* ELISA results are displayed for the panels of SARS-CoV-2 patient samples from 2020 (n = 68) and pre-SARS-CoV-2 negative controls with rS, rS-T4 and rRBD. Samples diluted 1/2000 was run in duplicate and the mean Abs 490 nm is displayed. Positive signal cut-off (*dashed line*) is the negative control sample mean + 3 SDs. E, Correlation plots of the Abs 490 nm data obtained for the SARS-CoV-2 patient samples from 2020 with rS-GFP and rS (*left*), rS and rS-T4 (*middle*), and rS and rRBD (*right*) are displayed with Pearson’s correlation coefficient (R). The rS-GFP data is from Figure 4*A*. *F,* percentage of the SARS-Cov-2 patient samples from 2020 that reacted with rS, rS-T4 and rRBD are displayed. Positive samples possessed an Abs 490 nm greater than the positive signal cut-off of the negative control samples. RBD, recombinant receptor binding domain; ELISA, enzyme-linked immunosorbent assay.

rS-GFP contains GFP and a Strep Tag on the *C*-terminus that could potentially alter the structure and hence the antibody reactivity. Therefore, we produced and purified a recombinant prefusion stabilized full-length S (rS) without the GFP and two additional S constructs commonly used in ELISAs (Fig. 5*A*). These included a secreted S chimera (rS-T4) where the *C*-terminus containing the TM domain was replaced with the T4 trimerization domain (43), and a secreted version of the S receptor binding domain (rRBD). We also produced WT recombinant full-length S (rS^WT^) without the prefusion stabilizing mutations for comparison (Fig. S6). All the constructs were expressed in insect cells using BVs and the proteins were purified either from the cells (rS and rS^WT^) or the culture medium (rS-T4 and rRBD). The isolated proteins showed bands at the expected molecular weights on non-reducing SDS-PAGE gels with other smaller species that were more prevalent under reducing conditions (Fig. 5*B*). SEC profiles indicated rS and rS-T4 formed high molecular weight (*e.g.,* > 690 kDa) structures like rS-GFP, whereas the major rRBD peak corresponded with a monomer (Fig. S7). In the StrepTactin ELISA format, rS-GFP, rS, rS-T4 and rRBD were recognized by a monoclonal antibody (MAb) against RBD and all but the rRBD were also recognized by MAbs against the *N-*terminal domain (NTD) and S2 region (Fig. 5*C*), indicating the proteins contained the expected epitopes in similar conformations.

We then compared the reactivity of the SARS-CoV-2 patient samples from 2020 with the recombinant S constructs and observed a wide range of reactivity (Fig. 5*D*), including with rS^WT^ (Fig. S8*A*). The rS results showed a strong linear correlation with those previously obtained with rS-GFP (Fig. 5*E*, left panel), indicating the GFP did not significantly alter the recognition of S by the antibodies in the samples. The rS results also correlated equally well with those obtained with the secreted rS-T4, but less so with the rRBD (Fig. 5*E*, middle and right panels) and rS^WT^ (Fig. S8*B*). Based on the negative control samples, we considered 41 test samples to be positive for rS, 38 for rS-T4, and 26 for rRBD (Fig. 5*F*). Of note, 34 out of 36 samples positive for rS-GFP (Fig. 3*F*) were positive with both rS and rS-T4, and the 26 rRBD positive samples were positive in all the ELISAs. Interestingly, the number of positive samples increased to 56 with rS^WT^ (Fig. S8*C*), suggesting several individuals developed antibodies that specifically recognized epitopes presented by rS^WT^.

### Correlation of the ELISA results with neutralization

**Figure 6:**
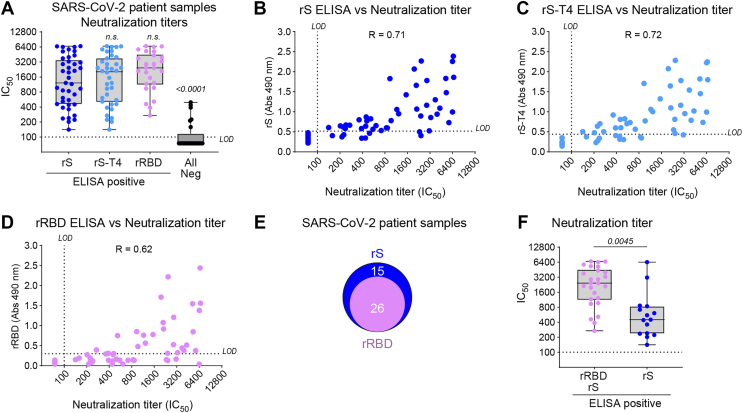
**Correlation between the recombinant S protein ELISA results and neutralization.***A,* box and whiskers plot showing the median and 25% quartiles of S pseudovirus neutralization titers measured using the ELISA positive rS, rS-T4, or rRBD SARS-CoV-2 patient samples from 2020. Neutralization titers of the ELISA negative samples are included as a control. Neutralization titers are displayed as median inhibitory concentrations (*IC*_*50*_). Limit of detection (LOD) is indicated. Significant and not significant (n.s.) *p* values are from a One-Way ANOVA using Dunnett’s multiple comparisons test with rS as a comparator. *B and D,* Correlation plots showing neutralization titers of the SARS-CoV-2 2020 patient samples with respect to the ELISA absorbance results against (*B*) rS, (*C*) rS-T4 and (*D*) rRBD. Pearson’s correlation coefficient (R) is included in each plot. *E,* venn diagram showing the overlap in the number of positive SARS-CoV-2 patient samples from the ELISAs with rS and rRBD. *F,* box and whiskers plot showing the median and 25% quartiles of the S pseudovirus neutralization titers of the SARS-CoV-2 2020 patient samples positive by ELISA with rRBD and rS, or rS alone. Neutralization titers are displayed as *IC*_*50*_ values. The *p* value is from a two-tailed Student *t* test. ELISA, enzyme-linked immunosorbent assay; rRBD, recombinant receptor binding domain.

To determine how the ELISA results correlated with neutralization titers we tested the SARS-CoV-2 patient samples from 2020 using pseudotyped particles that encode for the ancestral S protein. Measurable neutralization titers were found for all samples that were positive using rS, rS-T4 and rRBD (Fig. 6*A*), indicating none of the ELISAs identified false positive samples. The median neutralization titer was lower for the higher number of rS positive samples than rS-T4 and rRBD but there was no statistically significant difference between them. In contrast, ELISA negative samples showed significantly lower neutralization titers (*p* < 0.0001). For all three ELISAs, higher neutralization titer samples tended to produce higher absorbance readings and the rS and rS-T4 ELISAs showed slightly better linear correlations (Fig. 6, *B*–*D*), likely because these identified more samples that contained antibodies targeting regions outside the RBD. Supporting this conclusion, the rS^WT^ ELISA identified all samples with measurable pseudovirus neutralization activity (Fig. S8*D*). However, the rS^WT^ ELISA also identified several samples (n = 7) without neutralization activity, suggesting that some of the epitopes presented by rS^WT^ are recognized by non-neutralizing antibodies. Finally, we compared the neutralization titers of the 26 rRBD and rS positive samples *versus* the 15 rS positive samples (Fig. 6, *E* and *F*). Interestingly, samples that reacted with rRBD and rS showed significantly higher neutralization titers than the rS reactive samples (Fig. 6*F*), suggesting the ELISA data can be combined to identify high and low neutralization titer samples, respectively. The results from this proof of concept study provide a blueprint for producing recombinant viral envelope proteins with their TM domain intact that can be used in an ELISA for measuring antibody responses.

## Discussion

Antibody responses against envelope virus antigens are typically measured by ELISA using recombinant envelope proteins lacking the TM domains. As TM domains can influence membrane protein folding and assembly (24, 25, 26, 27, 28), we hypothesized that recombinant viral envelope proteins containing the TM domain are well suited for measuring functional antibodies that recognize native antigen structures. To test our hypothesis, we developed an approach for producing recombinant full-length versions of several envelope proteins on SARS-CoV-2. These included a prefusion stabilized version of the spike (rS-GFP), as well as the membrane (rM-GFP) and envelope (rE-GFP) proteins. The strategy involved expressing each protein as a *C*-terminal fusion with GFP to facilitate detection and a Strep-tag for purification. We then established an ELISA using the Strep-tag to display the recombinant proteins and the GFP fluorescence for coating consistency. Tests with different panels of adult human samples showed a range of rS-GFP reactivity with marginal rM-GFP and rE-GFP reactivity, supporting reports that S is more immunogenic than M and E (18, 40). We obtained similar results with recombinant prefusion stabilized S (rS) lacking GFP and included a WT full-length rS (rS^WT^) without the stabilizing substitutions for comparison. The ELISAs with rS and rS^WT^ identified more S-positive samples than a rRBD ELISA and all the rS reactive samples neutralized S-pseudotyped particles, indicating the rS ELISA produced fewer false negatives. Finally, we found that the rS and rRBD ELISA data can be combined to identify samples with high and low neutralization titers.

Creating an efficient production process for the SARS-CoV-2 envelope proteins significantly benefitted from adding GFP. First, GFP fluorescence revealed that all three membrane proteins are susceptible to insect cell proteases, which can increase during BV infection (44). Second, we used a simple GFP cleavage assay to identify appropriate inhibitors during cell lysis (*e.g.,* leupeptin), suggesting one or more serine or cysteine proteases encoded by insect cells is responsible (45, 46). Third, fluorescence-based protein integrity data indicated small amounts of protease also co-purified with the proteins and this was addressed by adding the covalent inhibitor PMSF to the lysate. Fourth, GFP fluorescence enabled quantification of bound antigen amounts with a standard plate reader, providing controls for increased consistency and reproducibility. Finally, the observation that all three recombinant membrane proteins were cleaved suggested this is a common problem in BV insect cell expression systems that can likely be addressed by identifying the proteases and using approaches to reduce or remove them.

With all the constructs we were able to isolate milligram quantities of protein per liter and ∼55% of the SARS-CoV-2 patient sera from 2020 reacted with the prefusion stabilized rS-GFP, which is in line with previous reports from early pandemic samples (42, 47). Somewhat expected, the percentage of rS-GFP reactive samples increased to ∼85% with the more recent healthy volunteer sera from 2022 to 2024 that were not prescreened for SARS-CoV-2 exposure. While the number of samples we analyzed was not extensive, the results suggest that antibodies against the prefusion S protein have become more prevalent in adults over time likely due to increased SARS-CoV-2 antigen exposure either by infection or vaccination.

In contrast, none of the sample panels significantly reacted with rM-GFP or rE-GFP even with a pan Ig secondary. It is equally possible that these smaller integral membrane proteins with a minimal ectodomain are significantly less immunogenic than S or that the GFP interferes with the native assembly of these smaller oligomers. However, we did not test the latter by removing the GFP due to the lack of specific monoclonal antibodies or established positive E and M sera for comparison. Additionally, it is not clear if the reported linear epitopes in M at positions 1 to 20 and 201 to 222 (18, 23) are recognized in the context of the full-length protein. Nevertheless, constructs with and without the GFP should be compared when developing a similar assay to test for potential negative impacts from adding GFP.

The correlation between rS-GFP to full-length rS and rS-T4 indicated that the GFP likely had minimal impact on the conformation and antibody recognition of the recombinant S constructs. To investigate how the prefusion stabilizing substitutions potentially impacted the results, we also analyzed the SARS-CoV-2 patient sera from 2020 with full-length rS^WT^. Interestingly, a higher percentage (∼82%) of the 2020 patient sera reacted with rS^WT^. This included 8 samples with measurable neutralization titers that were undetected with rS-GFP and rS as well as 7 samples with no measurable neutralization activity. We speculate the differences are attributed to mixed populations in the rS^WT^ preparation that include a prefusion S conformation that more closely resembles WT and another population in a post-fusion-like conformation or with different post-translational modifications as two bands of similar size were observed following isolation. While additional studies are needed to investigate this possibility, combining ELISAs with rS and a construct with a post-fusion conformation could provide valuable insight on the quality of the antibody responses against S and how it correlates with clinical outcomes, as well as the stability of the prefusion S constructs used in vaccines. For example, a comparison of the rRBD and rS data indicated that ∼25% of the SARS-CoV-2 patients from 2020 predominantly generated antibodies against the NTD or S2 region of S and these samples generally contained low neutralizing activity, whereas those that were rRBD and rS positive generally possessed higher neutralization titers. However, we did not test if these findings also apply to current samples or different S/RBD variants.

Overall, this proof of concept study provides a foundation for producing recombinant viral envelope proteins with their TM domains and using them in an ELISA to measure antibody responses. Our results with the full-length rS protein show that there are no clear reasons for avoiding using recombinant envelope proteins with the TM domain included. This is likely to be even more true for antigens where less structural and empirical data are available for identifying stable domains or producing secreted versions. To test this rationale, we also produced rS^WT^ and provide data indicating it is potentially a more sensitive antigen for detecting S-positive samples than prefusion stabilized versions and can likely provide more information when the results from the two constructs are combined. Future studies applying this approach are warranted to determine if this strategy is generally transferable to other viral envelope proteins and more broadly applicable for producing recombinant antigens for vaccines against enveloped viruses.

## Experimental procedures

### SARS-CoV-2 S, M and E gene constructs

Insect cell codon optimized genes encoding for the S (Gene ID: 43740568), M (Gene ID: 43740571) and E (Gene ID: 43740570) proteins from the ancestral SARS-CoV-2 strain (Wuhan/Hu-1/2020) were all synthesized (GenScript). All sequences included a 3′ region encoding a linker, a TEV protease site, the monomeric GFP from Olindias Formosa (48) followed by a Strep-tag II (30) and were inserted into a pFastBac1 vector using *Bam*HI (5′) and *Hind*III (3′) restriction sites. The rS-GFP construct contained these additional modifications: the S signal peptide was replaced by the azurocidin signal peptide to increase insect cell expression (49), K986P and V987P substitutions to stabilize the prefusion conformation and GSAS substitutions at residues 682 to 685 to remove the RARR furin cleavage (22, 36). The rS construct was created by removing the linker, TEV site and the GFP from the rS-GFP construct. rS^WT^ was generated by reintroducing WT residues K986, V987, R682, A683, R684 and R685 back into rS. The secreted rS-T4 construct was generated by fusing the bacteriophage T4 trimerization domain (43) and a Strep-tag II (ST) to residues 1213 in the rS construct. The rRBD construct contained the azurocidin signal peptide followed by residues 319 to 541 from the S protein (50) and a ST.

### Generation of recombinant baculoviruses

Recombinant BV for each protein were produced using the Bac-to-Bac Baculovirus Expression system (Gibco). Each pFastBac1 vector (200 pg) was used to transform 20 μl MAX Efficiency DH10Bac *E. coli* competent cells (Gibco) by heat shock at 42 °C for 45 s followed by ice (2 min), addition of SOC (180 μl) and recovery in a 37 °C Southwest Science SH1012 shaking incubator (225 rpm) for ∼4 h. *E. coli* were serially diluted and plated on LB agar plates containing 7 μg/ml gentamicin, 50 μg/ml kanamycin, 10 μg/ml tetracycline, 100 μg/ml X-gal and 40 μg/ml isopropyl-β-D-thiogalactoside and incubated at 37 °C for ∼48 h. White colonies were re-streaked and insertions were confirmed by PCR screening using pUC/M13 Forward (5′-CCCAGTCACGACGTTGTAAAACG-3′) and Reverse (5′-AGCGGATAACAATTTCACACAGG-3′) primers that hybridize sites flanking the mini-attTn7 in the *lacZ* complementation region of the bacmid containing the BV genome. Selected clones (expected size of insert + ∼2300 bp) were grown overnight in LB containing 7 μg/ml gentamicin, 50 μg/ml kanamycin and 10 μg/ml tetracycline in a 37 °C shaking incubator. Bacmid DNA was isolated with a BAC DNA miniprep kit (Zymo Research) and 12.5 μg DNA was mixed with 30 μl ExpiFectamine SF transfection reagent (Gibco) in 1 ml Opti-MEM I Reduced Serum Medium (Gibco) and added to 25 ml of Sf9 cells diluted to 2.5 × 10^6^ cells/ml in ExpiSf CD Medium (Gibco). Cells were incubated in a Brunswick Excella E24 (E24) shaking incubator (125 rpm) at 27 °C for 4 to 5 days and the P0 BV progeny was harvested by retaining the culture medium following centrifugation (4000*g*; 10 min). Working BV stocks (P1) were produced by infecting Sf9 cells in Sf-900 III SFM at a density of ∼2.5 × 10^6^ cells/ml with the P0 BV (0.2% V/V) for 4 to 5 days and collecting the clarified culture medium. Infections were evaluated by GFP fluorescence in the cells and culture medium and fluorescence microscopy.

### Fluorescence measurements in cells and culture medium

Sf9 cells were grown in the dark with Sf-900 III SFM containing Penicillin (50 U/ml) and Streptomycin (50 μg/ml) using a 27 °C E24 shaking incubator (125 rpm) and split to ∼3 × 10^6^ cells/ml when the cell density reached 2 to 4 × 10^6^ cells/ml. For infections, Sf9 cells at a density of ∼2 × 10^6^ cells/ml were infected with 2% (V/V) of recombinant BVs (P1) and GFP fluorescence was monitored in cells and medium every 24 h. Briefly, culture samples (250 μl) at each time point were sedimented (15,000*g*; 1 min), culture medium was collected, and cell pellets were resuspended in 500 μl PBS pH 7.2. Both samples (100 μl each) were transferred to 96-well black clear bottom plates and the fluorescence (Ex λ 485 nm/Em λ 528 nm) was measured using a Cytation 5 plate reader (Biotek).

### Fluorescence microscopy

BV-infected Sf9 cells (250 μl) were collected, sedimented (15,000*g*; 1 min) and resuspended in 500 μl PBS pH 7.2. Resuspended cells (20 μl) were mixed with 80 μl PBS pH 7.2 containing 10 μg/ml Hoechst (Invitrogen). Cells were transferred to a flat-bottom 96-well plate (Falcon) and visualized using a BZ-X800 all-in-one Fluorescence microscope (Keyence) with a PlanFluor objective and BZ-X cube filter for Hoechst (Ex λ 360 nm/Em λ 460 nm) and GFP (Ex λ 470 nm/Em λ 525 nm). Images were analyzed using BZ-II Analyzer software (Keyence) and cropped with Adobe Photoshop.

### SDS-PAGE analysis

Recombinant proteins were resolved on 8 to 16% Novex Tris-Glycine SDS-PAGE WedgeWell gels (Thermo Fisher Scientific). Clarified cell lysates or purified proteins (∼2.5 μg) were mixed with equal volume of 2× Tris-Glycine SDS sample buffer and directly loaded onto the gel, or with sample buffer containing 100 mM dithiothreitol (DTT) and heated at 95 °C for 5 min prior to loading. Gels were run at 150V constant voltage for 1 h, washed with dH_2_O and imaged with an Azure C600 Bioimager (Ex λ 472 nm/Em filter λ 513 nm) for in-gel fluorescence. Coomassie gels were washed three times in dH_2_O, stained with Simply blue (Thermo Fisher Scientific) and imaged directly. Novex Sharp Unstained protein standard (Thermo Fisher Scientific) was included for molecular weight references.

### Size exclusion chromatography

Size exclusion chromatography was performed using an Agilent 1260 prime HPLC equipped with an autoloader, variable wavelength detector (VWD)and an AdvanceBio SEC 300A column (Agilent Technologies) equilibrated with 50 mM Tris-HCl pH 7.0 containing 150 mM NaCl and 0.01% Triton X-100 (TX-100). Samples (20 μl) were analyzed using 1 ml/min flow rate and the absorbance (Abs) at 260 nm and 280 nm were monitored with the VWD, and fluorescence (Ex λ 485 nm/Em λ 528 nm) with the fluorescence detector. For analysis of rS-GFP, rS, rS-T4 and rRBD with the VWD, samples (20 μl of ∼0.2 mg/ml) were run using the equilibration buffer and running parameters. AdvanceBio SEC 300A protein standard (Agilent) was used for molecular weight references.

### Recombinant protein purification

BV infected Sf9 cells (2% V/V) were collected 48 to 72 h post-infection by centrifugation (4000*g*; 10 min). Cell pellets were resuspended in Buffer A (50 mM Tris buffer pH 7.0 containing 150 mM NaCl, 10 U/ml Benzonase Nuclease (EMD Millipore Corporation), 1 mM MgCl_2_, 1 mM PMSF and 1× protease inhibitor (PIN) cocktail (Sigmafast protease inhibitor cocktail tablets)). Cell membranes were disrupted using three passes through an Avestin EmulsiFlex-C3 at 5 to 10,000 psi, membrane fractions were isolated by sedimentation (125,000*g*; 60 min), solubilized with Buffer A containing 2% TX-100 (∼one-third original culture volume), rotated overnight at 4 °C and insoluble material was sedimented (125,000*g*; 60 min). Clarified supernatant was loaded onto a 5 ml StrepTactin XT 4Flow resin (IBA Lifesciences) column, washed with 15 column volumes (CVs) of Buffer B (50 mM Tris pH 7.0, 150 mM M NaCl, 0.1% TX-100) and proteins were eluted in 4 ml fractions using 10 CVs Buffer B containing 50 mM biotin. Fractions with the highest GFP fluorescence were pooled and dialyzed three times against Buffer B at 4 °C using a Slide-a-lyzer dialysis cassette (Thermo Fisher Scientific) with a 20 kDa molecular weight cut-off. Fractions for proteins without GFP were identified by the Abs 280 nm. Protein concentration was measured with a Micro BCA Protein Assay Kit (Thermo Fisher Scientific), aliquoted and stored at −80 °C.

### ELISA optimization with StrepTactin-coated plates

Purified proteins were serially diluted in Coating Buffer (150 mM Tris-HCl containing pH 7.0, 150 mM NaCl and 0.01% TX-100), transferred (100 μl/well) to StrepTactin-coated microplate wells (IBA Lifesciences), and fluorescence (Ex λ 485 nm/Em λ 528 nm) was measured using a Cytation 5 plate reader. Plates were incubated 2 h at 37 °C, washed (3 × 200 μl) with PBS-TXG (PBS pH 7.2 containing 0.001% TX-100 and 1% normal goat serum) and bound protein was measured in the last wash by fluorescence. SARS-CoV-2 Positive (WHO neutralization assay standard [WHO IS 21-338, NIBSC, UK]) and Negative pooled human serum (Innovative Research, cat # IPLA-SER) controls were serially diluted in PBS-TXG, transferred to the plate (100 μl/well), and incubated 1 h at 37 °C. For an additional positive control, convalescent serum from K-18 mice infected with the ancestral SARS-CoV-2 strain was compared to negative serum from naïve K-18 mice. Plates were washed (3 × 200 μl) with PBS-TXG and incubated 30 min at 37 °C with a HRP-conjugated rabbit anti-human IgG (Invitrogen) diluted 1:20,000 in PBS-TXG. Plates were washed (3 × 200 μl) with PBS-TX (PBS pH 7.2 containing 0.001% TX-100) and developed using *o*-phenylenediamine dihydrochloride (Sigma) for 10 min at room temperature. Reactions were stopped with 1N H_2_SO_4_ (100 μl/well) and the Abs 490 nm was read on a Cytation 5 plate reader.

### Human sera analysis by ELISA with StrepTactin-coated plates

Deidentified human plasma samples (n = 68) from lab-confirmed SARS-CoV-2 cases (acute and convalescent) collected between 4/21/2020 and 5/18/2020 were obtained from Washington Adventist Medical HealthCare and inactivated by heating at 56 °C for 60 min. Study of these samples was given exempt status approval from the U.S. Food and Drug Administration’s Research Involving Human Subjects Committee. Deidentified negative control pre-pandemic human sera (n = 16) collected between 2013 to 2014 were from a previous study (51). Post-pandemic human sera (n = 19) collected between 2022 and 2024 were from recent healthy volunteer studies performed at the NIH Clinical Center after participants signed an informed consent form. Purified proteins were diluted in Coating Buffer to 8 μg/ml and added (100 μl/well) to StrepTactin-coated microplates. Plates were incubated 2 h at 37 °C and washed (3 × 200 μl) with PBS-TXG. Plasma/sera samples were diluted 1:2000 in PBS-TXG and transferred (100 μl/well) to the Strep-Tactin-coated microplate. Plates were incubated 1 h at 37 °C and processed as described in ELISA optimization. The positive signal cut-off corresponded to the average of the 16 pre-pandemic samples on each plate plus 3 SDs.

### ELISA monoclonal antibody analysis

MAbs to the S protein *N-*terminal domain (NTD), S2 domain and receptor binding domain (RBD) were obtained from Biointron. Proteins were diluted in Coating Buffer to 8 μg/ml, added (100 μl/well) to Strep-Tactin-coated microplates and incubated 2 h at 37 °C. Plates were washed (3 × 200 μl) with PBS-TXG. MAbs were serially diluted in PBS-TXG and transferred (100 μl/well) to the Strep-Tactin-coated microplate. Plates were incubated 1 h at 37 °C, washed (3 × 200 μl) with PBS-TXG and incubated 60 min at room temperature with a HRP-conjugated goat anti-mouse IgG (Invitrogen) diluted 1:2000 in PBS-TXG. Plates were washed (3 × 200 μl) with PBS-TX and developed using 1-Step TMB ELISA Substrate (Thermo Fisher Scientific) at room temperature. Reactions were stopped with 1N H_2_SO_4_ (100 μl/well) and the Abs 450 nm was read with a SpectraMax iD3 plate reader.

### ELISA analysis with secreted S produced from Expi293F cells

Serum samples were tested on Maxisorp 96-well plates (Nunc) coated overnight at 4 °C with soluble S protein (1 μg/ml) purified from Expi293F cells (Thermo Fisher Scientific) as previously described (52). Briefly, samples were diluted 1:100 in PBST (PBS pH 7.2 with 0.05% Tween-20) containing 5% skim milk, added to the plates and incubated overnight at 4 °C. Plates were washed with PBST, HRP-conjugated anti-mouse IgG (NXA9311ML, Cytiva) diluted 1:500 in PBST/5% milk was added and plates were incubated 60 min at room temperature. After washing with PBST plates were developed using ABTS peroxidase substrate (SeraCare) for 10 min at room temperature. Reactions were stopped with 1% SDS and the Abs at 405 nm was measured using a Synergy Plate Reader (Biotek).

### SARS-CoV-2 S pseudovirus neutralization assay

Preparation of the Lentivirus-based SARS-CoV-2 S pseudotyped particles and the neutralization assay have previously been described (52, 53). Briefly, neutralization assays were performed using 293T-ACE2/TMPRSS2 cells (54) and the half maximal inhibitory concentration (*IC_50_*) was calculated using the Reed and Muench method. Titers are expressed as the reciprocal sera dilution. Samples without an *IC*_*50*_ by the lowest dilution (1:200) were assigned 100.

### Statistical analysis

All analysis was performed using GraphPad Prism 10 software assuming the sample groups possess a Gaussian distribution and equal SDs. Student’s unpaired *t* test was performed between groups using a confidence interval of 95%. One-way ANOVAs were performed with Dunnett’s multiple comparisons test at a confidence interval of 95% to compare more than two related sample groups. Pearson's correlation analysis was used to measure the relationship of the ELISA data with different recombinant S proteins and to compare the ELISA and neutralization data. *p* values lower than 0.05 were considered significant.

## Data availability

All datasets are presented in the manuscript and supplementary material.

## Supporting information

This article contains supporting information.

## Ethics statement

The contents of this publication are an informal communication and represent the best judgment of the authors. These comments do not bind or obligate FDA. All animal experiments followed Protocol #2003-18 that is approved by the U.S. FDA Institutional Animal Care and Use Committee (IACUC). The animal care and use protocol meets the National Institutes of Health guidelines. Study of the deidentified human samples (n = 68) from SARS-CoV-2 patients (acute and convalescent) collected between 4/21/2020 and 5/18/2020 by Washington Adventist Medical HealthCare was given exempt status approval from the U.S. Food and Drug Administration’s Research Involving Human Subjects Committee. Pre-SARS-CoV-2 human sera (n = 16) collected between 2013 to 2014 and post-pandemic human sera (n = 19) from 2022-24 were from healthy volunteer studies performed at the NIH Clinical Center after participants signed an informed consent form.

## Conflict of interest

The authors declare that they have no conflicts of interest with the contents of this article.
