# Supplementary material for: Development of a recombinant membrane protein ELISA for analyzing antibody responses against SARS-CoV-2 envelope proteins
Source: J Biol Chem. 2025 Nov 25;302(1):110974. doi: 10.1016/j.jbc.2025.110974 (PMC12794509; doi:10.1016/j.jbc.2025.110974)
Supplement: Supplementary figures [file mmc1.pdf]

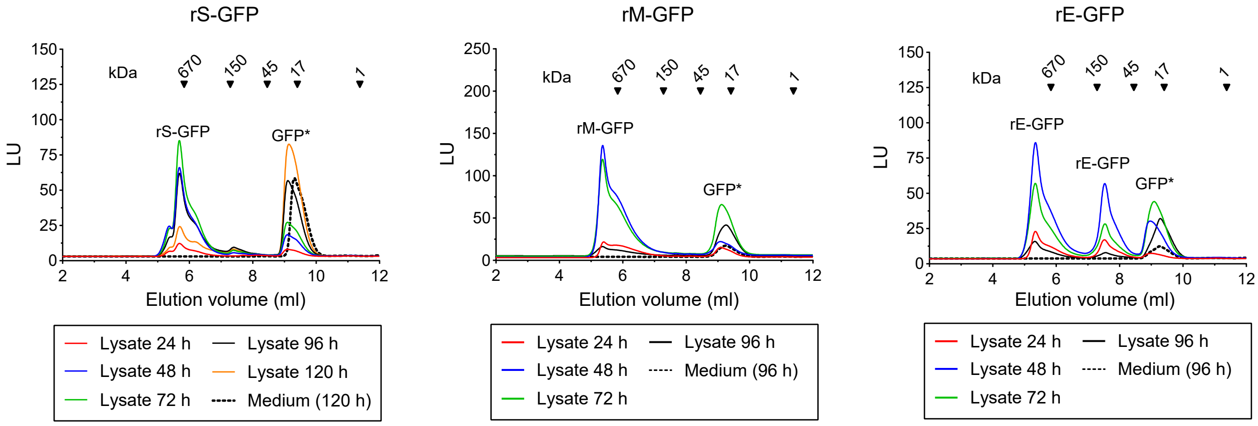

Supplemental Figure 1. FSEC profiles of insect cell lysates at the indicated times post-infection with BVs expressing rS-GFP, rM-GFP and rE-GFP. Cells sedimented from equal culture volume were lysed and clarified by sedimentation prior to FSEC analysis. Clarified culture medium was also analyzed. Luminescence units (LU) and molecular weight standards are included for reference. Peaks corresponding to full-length protein and GFP* are indicated.

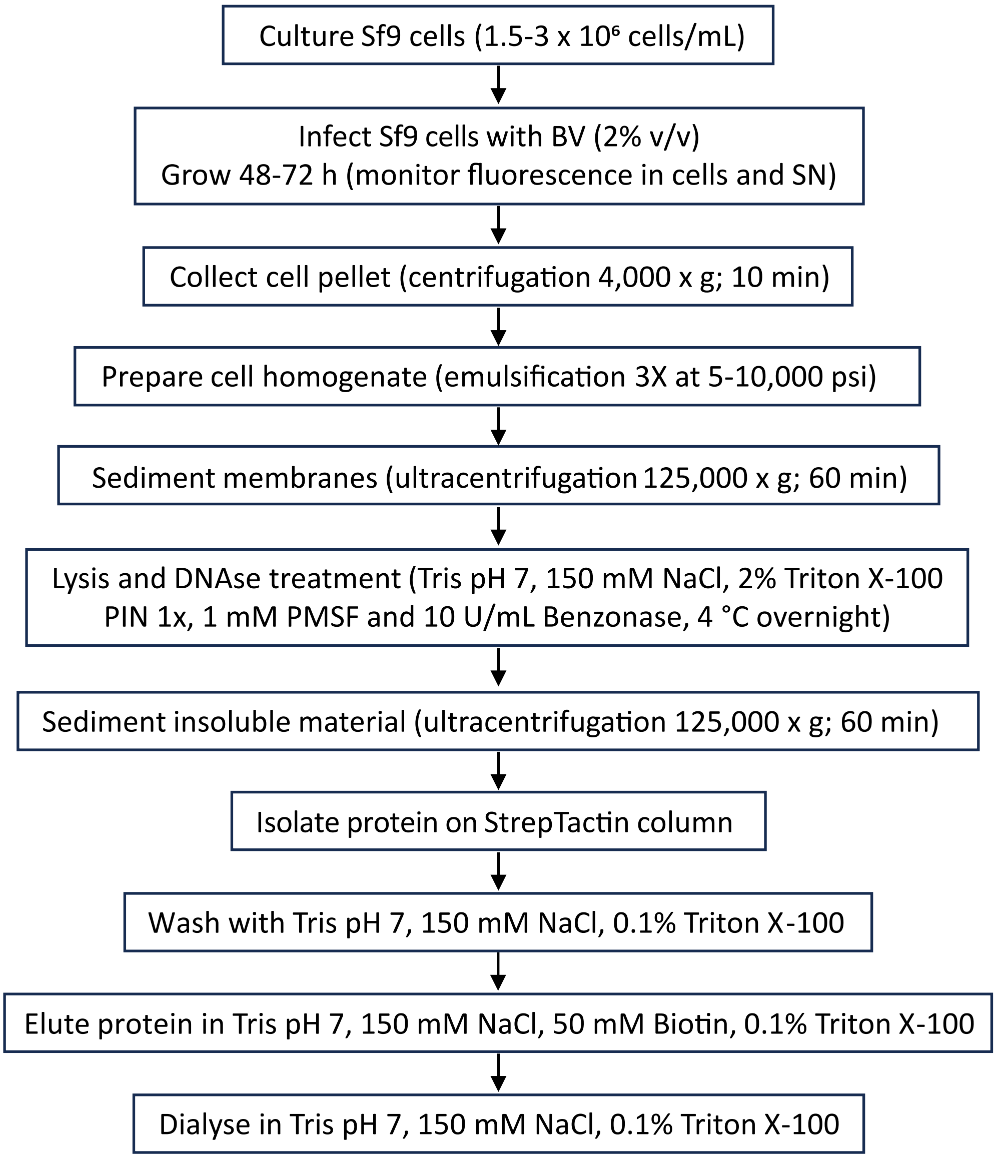

Supplemental Figure 2. Flow chart showing the expression and purification strategy for the recombinant SARS-Cov-2 envelope proteins fused to a C-terminal GFP and Strep-tag.

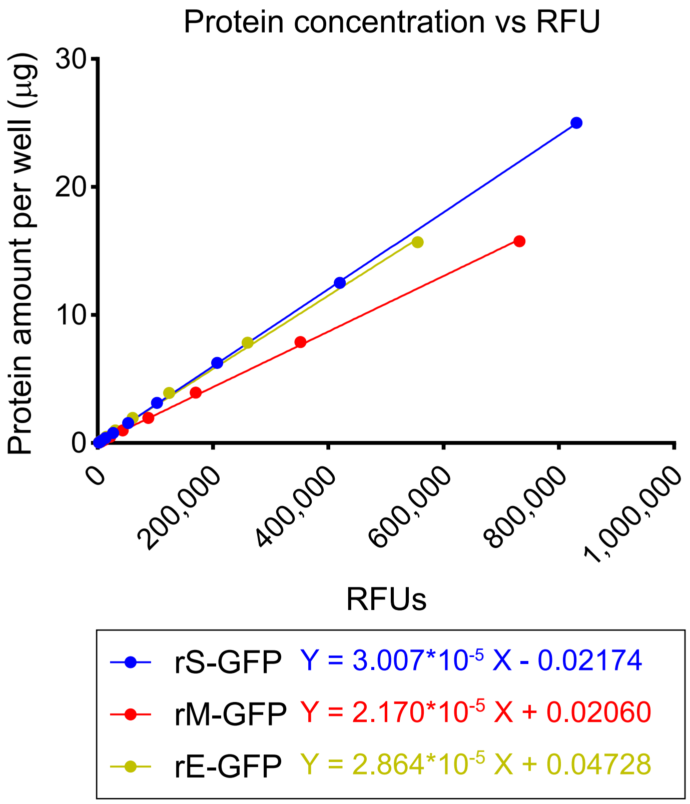

Supplemental Figure 3. Standard curves for calculating rS-GFP, rM-GFP and rE-GFP amounts based on fluorescence. Purified protein concentrations were measured with a Micro BCA assay, proteins were serially diluted and the relative fluorescence units (RFUs) for each well were plotted with respect to the total protein amount. Line equations for each standard curve are included.

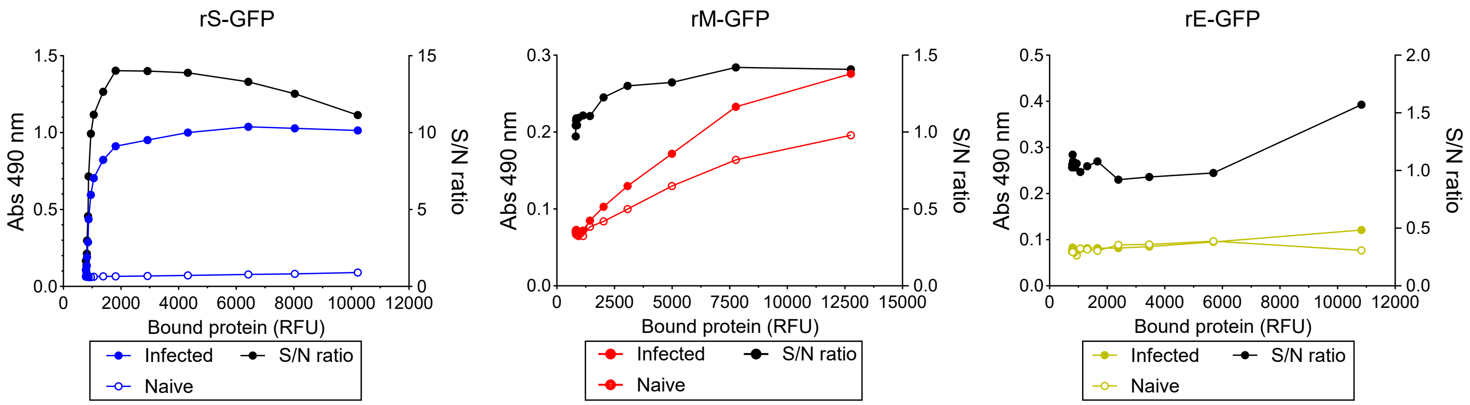

Supplemental Figure 4. Signal to noise (S/N) ratios are displayed from an ELISA performed with fixed dilutions (1:250) of pooled sera from SARS-CoV-2 (WA1) infected mice (positive control) and uninfected naïve mice (negative control) in wells with the indicated amounts of bound rS-GFP, rM-GFP and rE-GFP. Results are the mean from a representative experiment performed in duplicate.

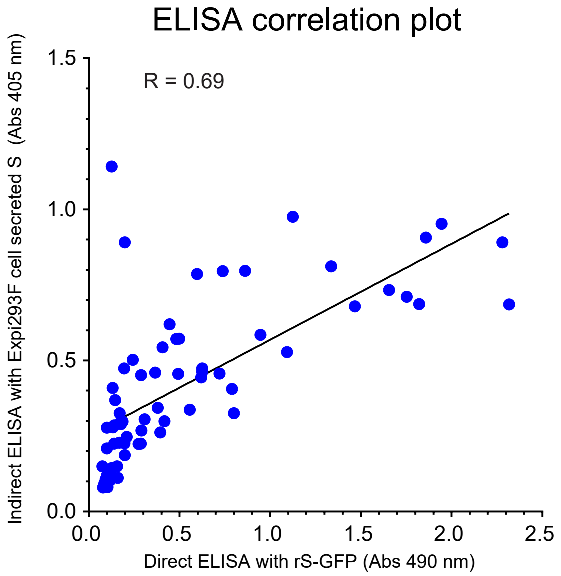

Supplemental Figure 5. Correlation plot showing absorbance values of the SARS-CoV-2 patient samples from 2020 with the rS-GFP ELISA versus an ELISA with a secreted rS protein produced in Expi293F cells directly adsorbed to the wells. Serum samples were diluted 1:100 for the direct ELISA and 1:2000 for the indirect rS-GFP ELISA. Pearson's correlation coefficient (R) is shown.

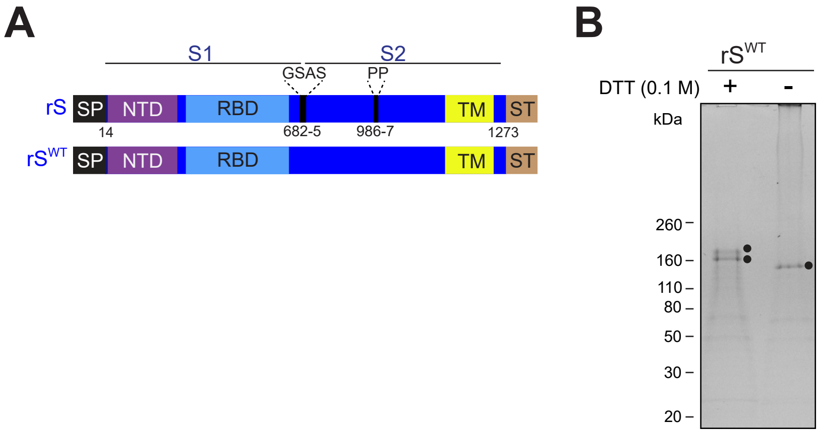

Supplemental Figure 6. Production of wild type recombinant full-length S. **A.** Diagram of the BV expression constructs encoding full-length prefusion stabilized S (rS) and wild-type S (rS^{WT}) from the Wuhan/Hu-1/2020 strain. Positions of the SP, ST and prefusion stabilizing mutations (GSAS and PP) are indicated. **B.** Representative Coomassie stained SDS-PAGE gel (8-16%) showing the purified rS^{WT} (circles). Samples were mixed with sample buffer and loaded directly or with sample buffer containing 0.1 M DTT and heated at 95 °C for 5 min prior to loading. Note under reducing conditions (+DTT) rS^{WT} resolved as two bands denoted by filled circles.

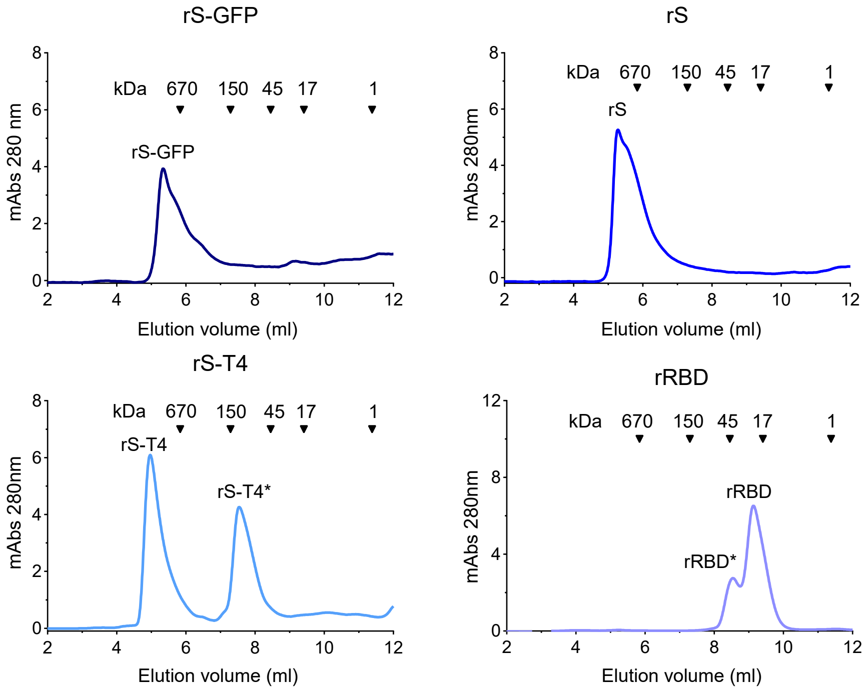

Supplemental Figure 7. SEC profiles of purified rS-GFP, rS, rS-T4 and RBD are displayed with molecular weight standards included for reference. Peaks corresponding to each full-length protein, an rRBD dimer (rRBD*), and an rS-T4 truncate (rS-T4*) are indicated.

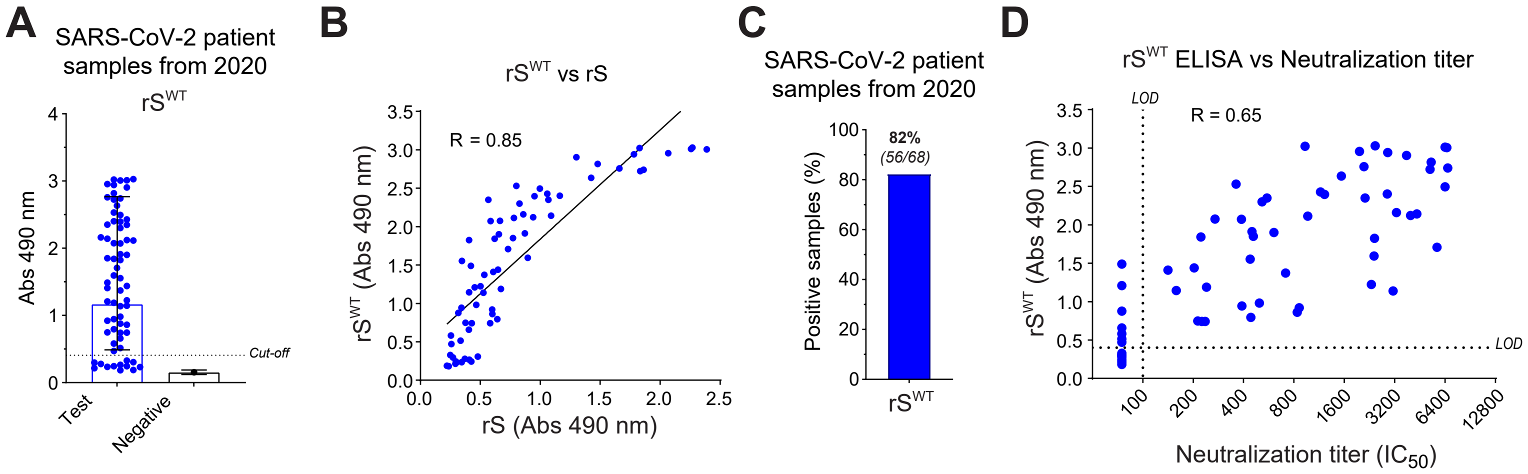

Supplemental Figure 8. Comparison of ELISA results from the prefusion stabilized rS and wild type full-length recombinant S (rS^{WT}). **A.** ELISA results are displayed for the panel of SARS-CoV-2 patient samples from 2020 (n=68) and a pre-SARS-CoV-2 negative control with rS^{WT}. Samples diluted 1/2000 were run in duplicate and the mean Abs 490 nm are displayed. Positive signal cut-off (dashed line) is indicated. **B.** Correlation plot of the Abs 490 nm data obtained for the SARS-CoV-2 patient samples from 2020 with rS^{WT} and rS is displayed with Pearson's correlation coefficient (R). **C.** Percentage of the SARS-Cov-2 patient samples from 2020 that reacted with rS^{WT} is displayed. **D.** Correlation plot showing the neutralization titers of the SARS-CoV-2 2020 patient samples with respect to the ELISA results against rS^{WT}. The R value is included.
